# Supplementary material for: Synthesis and Exon-Skipping Properties of a 3′-Ursodeoxycholic Acid-Conjugated Oligonucleotide Targeting DMD Pre-mRNA: Pre-Synthetic versus Post-Synthetic Approach
Source: Molecules. 2021 Dec 17;26(24):7662. doi: 10.3390/molecules26247662 (PMC8707236; doi:10.3390/molecules26247662)
Supplement: Supplementary file 1 [file molecules-26-07662-s001.zip › molecules-1482163-supplementary.pdf]

Supplementary Materials

Synthesis and Exon-Skipping Properties of a 3′-Ursodeoxycholic Acid-Conjugated Oligonucleotide Targeting *DMD* Pre-mRNA: Pre-Synthetic versus Post-Synthetic Approach

Elena Marchesi [1], Matteo Bovolenta [2], Lorenzo Preti [3], Massimo L. Capobianco [4], Kamel Mamchaoui [5], Monica Bertoldo [3,4] and Daniela Perrone [1,*]

[1] Department of Environmental and Prevention Sciences, University of Ferrara, 44121 Ferrara, Italy; mrclne@unife.it
[2] Department of Translational Medicine, University of Ferrara, 44121 Ferrara, Italy; bvlmtt@unife.it (Ma.B.)
[3] Department of Chemical, Pharmaceutical and Agricultural Sciences, University of Ferrara, 44121 Ferrara, Italy; prtlnz@unife.it (L.P.); brtmnc@unife.it (Mo.B.)
[4] Institute of Organic Synthesis and Photoreactivity-Italian National Research Council, 40129 Bologna, Italy; massimo.capobianco@isof.cnr.it
[5] Centre de Recherche en Myologie, Institut de Myologie, Sorbonne Université, Inserm, F-75013 Paris, France; kamel.mamchaoui@upmc.fr
* Correspondence: prd@unife.it

Table S1. Reaction conditions for formation of amide between UDCA and ASO 51 3′-C6NH_2.

Entry[a]	ASO Concentration (mM/Solvent)	UDC-NHS 1 (eq)	DIPEA (eq)	Time (h)	Yield[b] (%)
**1**	1.1 mM/ $NaHCO_3$, pH 8.5/DMSO 1:1	3	0	0.5	45
**2**	1.1 mM/ $NaHCO_3$, pH 8.5/DMSO 1:1	3	0	18	48
**3**	1.1 mM/ $NaHCO_3$, pH 8.5/DMSO 1:1	6	0	18	52
**4**	1.1 mM/ $NaHCO_3$, pH 8.5/DMSO 1:1	10	0	18	52
**5**	2.5 mM/ $NaHCO_3$, pH 8.5/DMSO 1:1	6	0	18	58
**4**	5 mM/ DMF	2	40	3	80
**5**	5 mM/ DMSO	2	40	18	80
**6**	5 mM/ DMSO	2	10	18	80

[a]All reactions were carried out at room temperatue. [b]Estimated conversion based on UV area of conjugated relative to unconjugated ASO 51 3′-C6-NH_2.

IP-HPLC chromatograms.

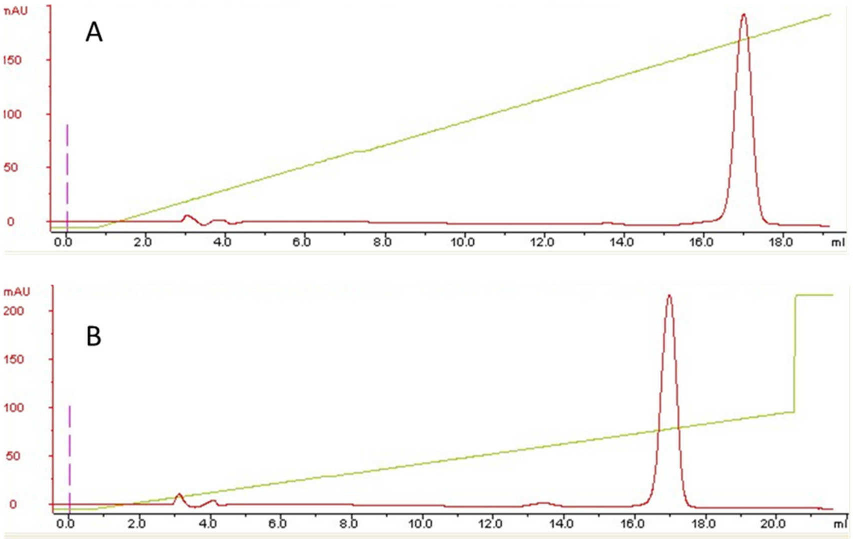

Figure S1. stability studies.

IP-HPLC (260 nm) chromatograms; A: reference ASO 51 3′-UDC; B: ASO 51 3′-UDC after deprotection/cleavage conditions. IP-HPLC analysis were executed on ÄKTA purifier with reverse phase column Resource RPC 3 ml using ion pairing buffer conditions: buffer A: pH= 8 triethylammonium acetate (TEAA) with 5% ACN; buffer B: ACN, working in acetonitrile gradient (ACN gradient: 15% in 2 CV, 45% in 4 CV).

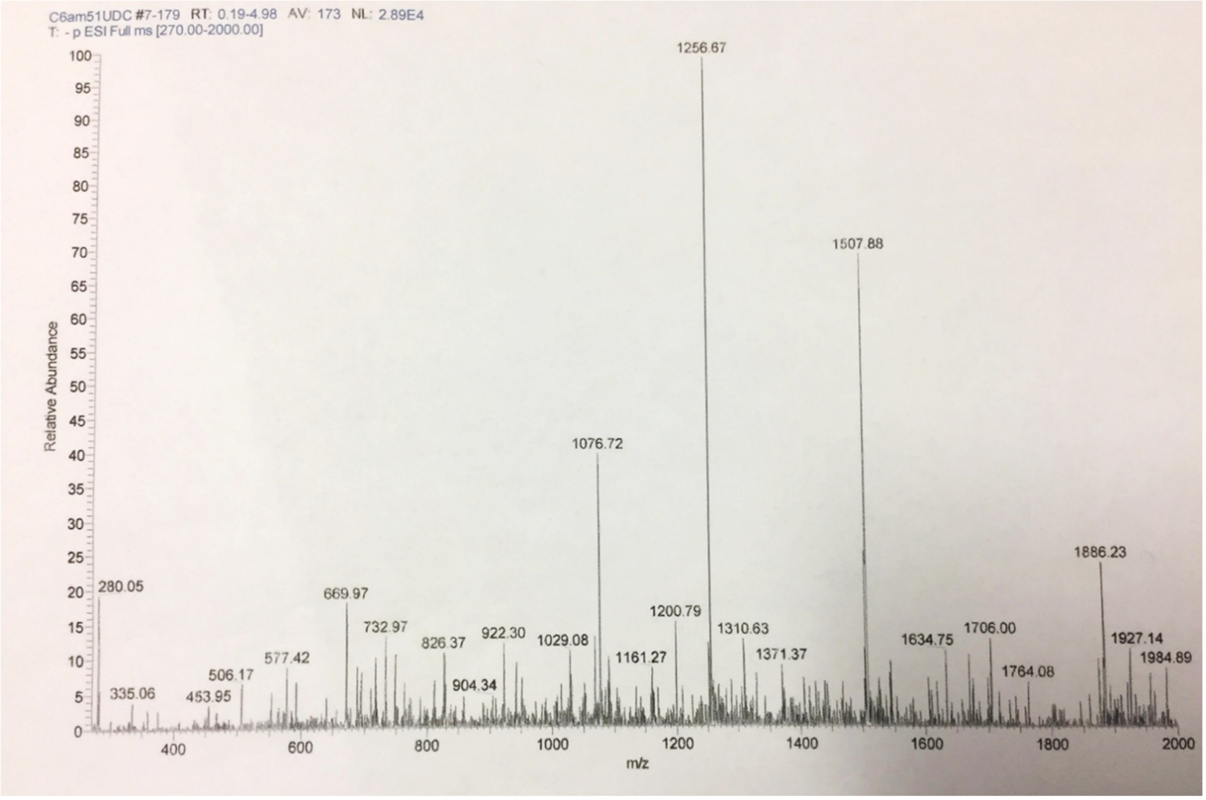

Figure S2. ASO 51 3′-UDCA mass spectrum.

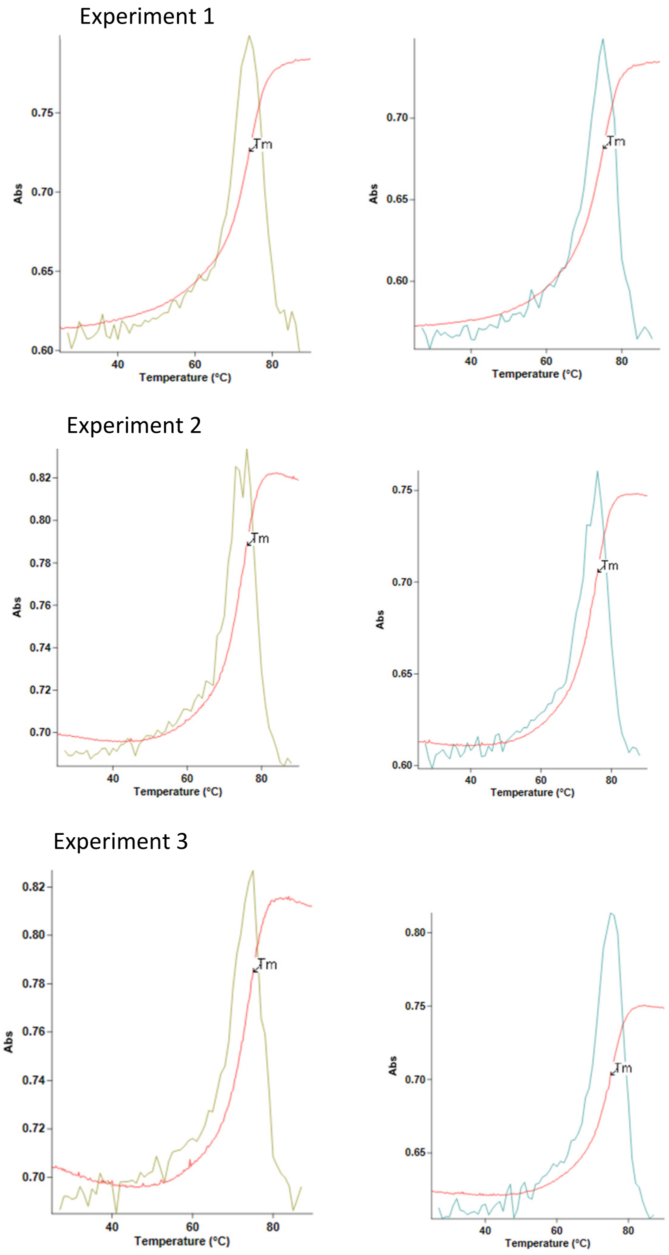

Figure S3. Melting curves.

Melting curves for naked ASO 51 (left) and ASO 51 3'-UDC (right).

Sample	T_m (°C)	T_m (K)
**ASO 51 (1)**	74.02	347.17
**ASO 51 (2)**	76.02	349.17
**ASO 51 (3)**	75.02	348.17
**ASO 51 3'-UDC (1)**	75.02	348.17
**ASO 51 3'-UDC (2)**	76.02	349.17
**ASO 51 3'-UDC (2)**	75.02	348.17

Spectra:

Compound 2: ***3-Hemisuccinyl-7-Acetyl-UDC-Amino-DMT-Hexanol***

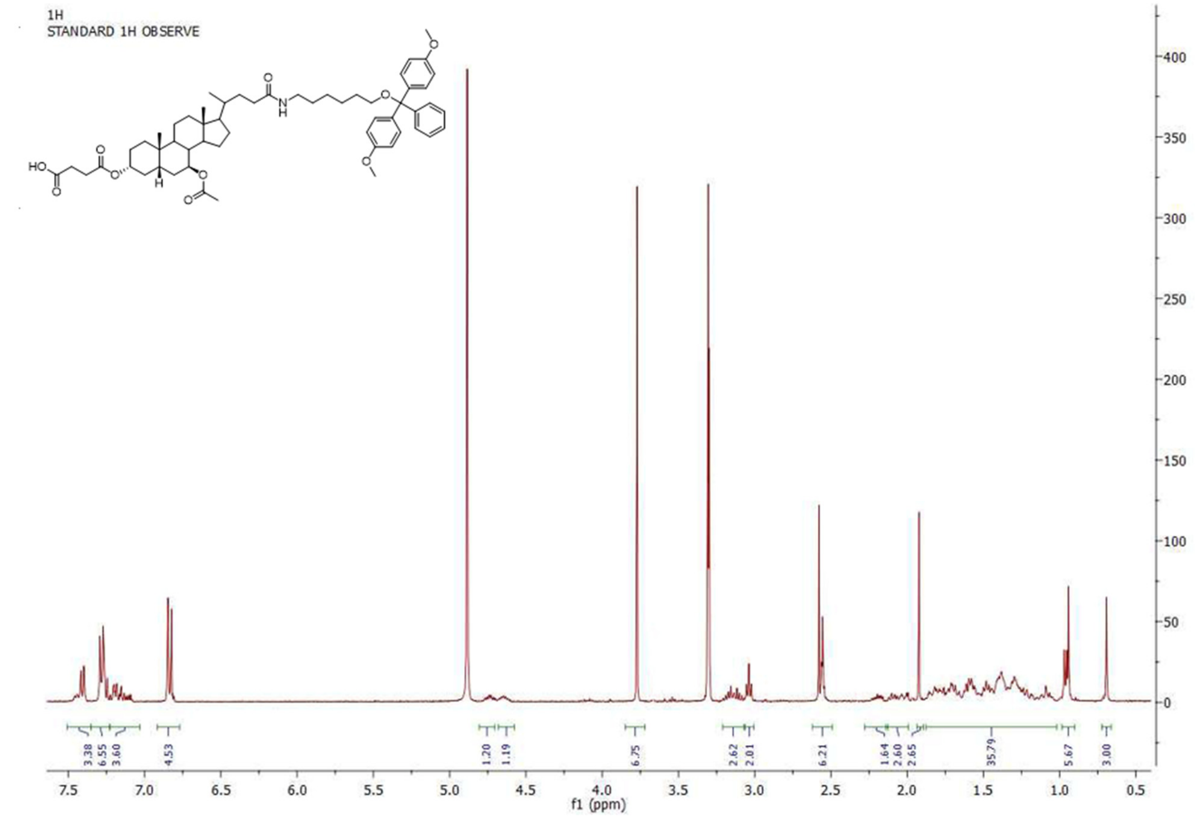

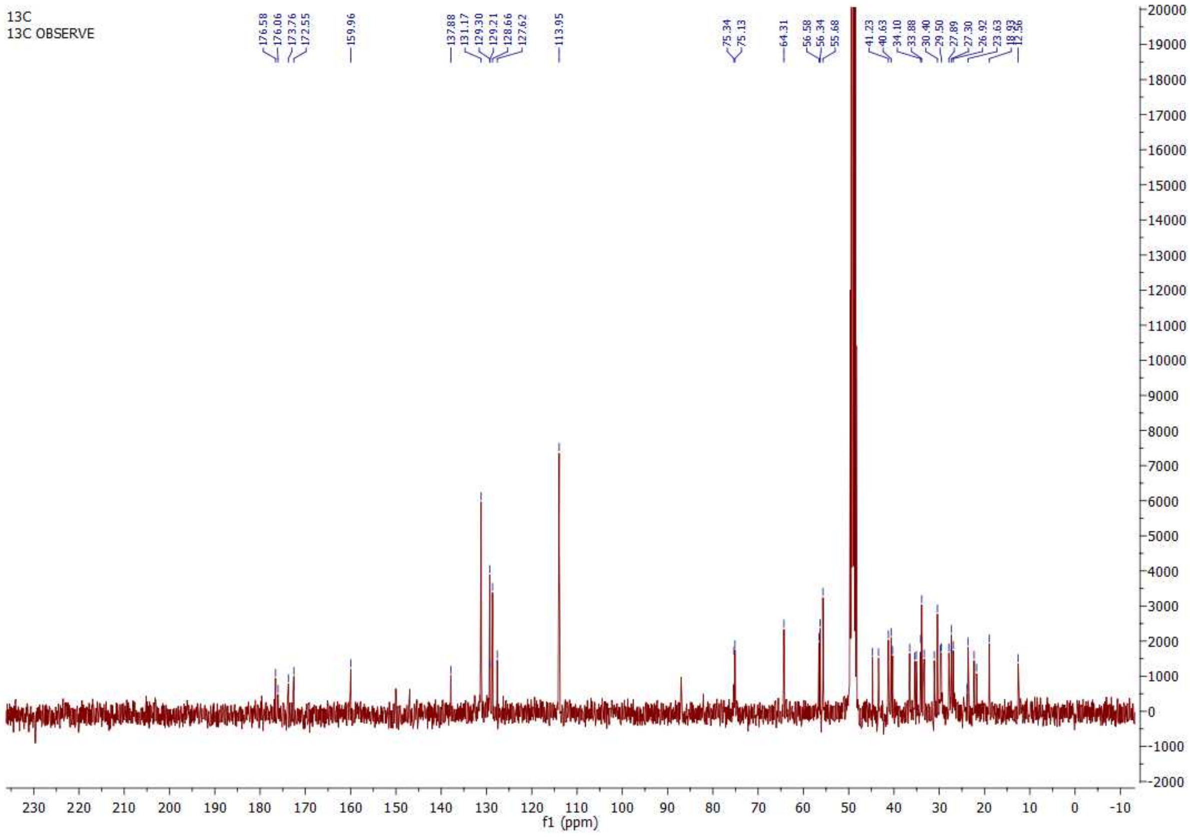

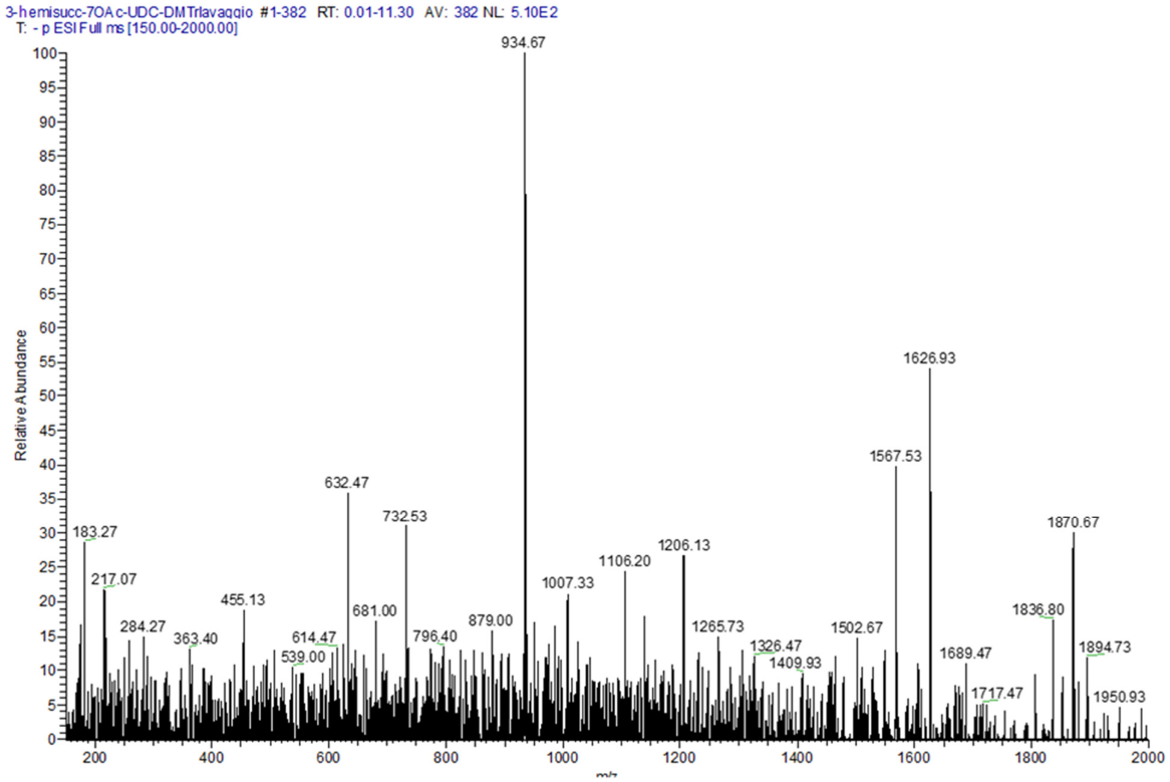

$[M\text{-}H]^-$: calculated for $[C_{57}H_{76}NO_{10}]^-$ 935.23; found 934.67.

Compound 6: ***7-Acetyl-UDC-Amino-DMT-Hexanol***

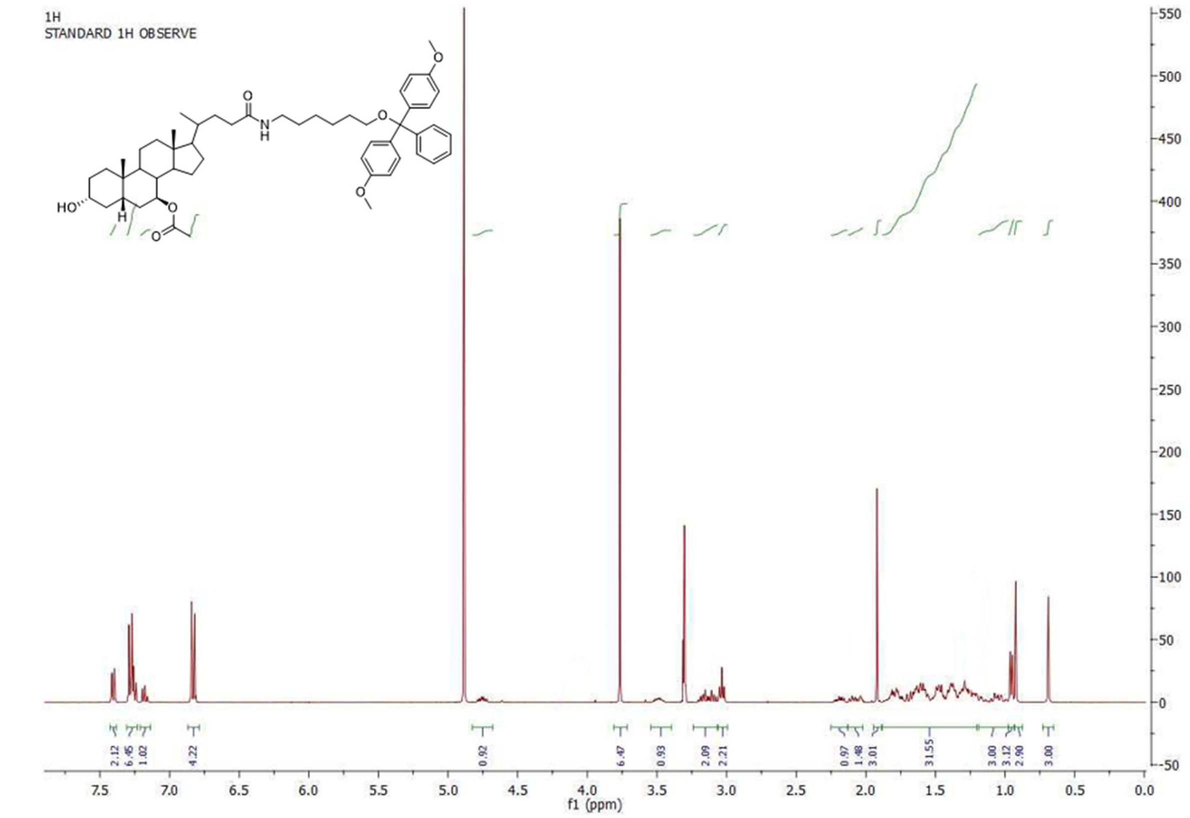

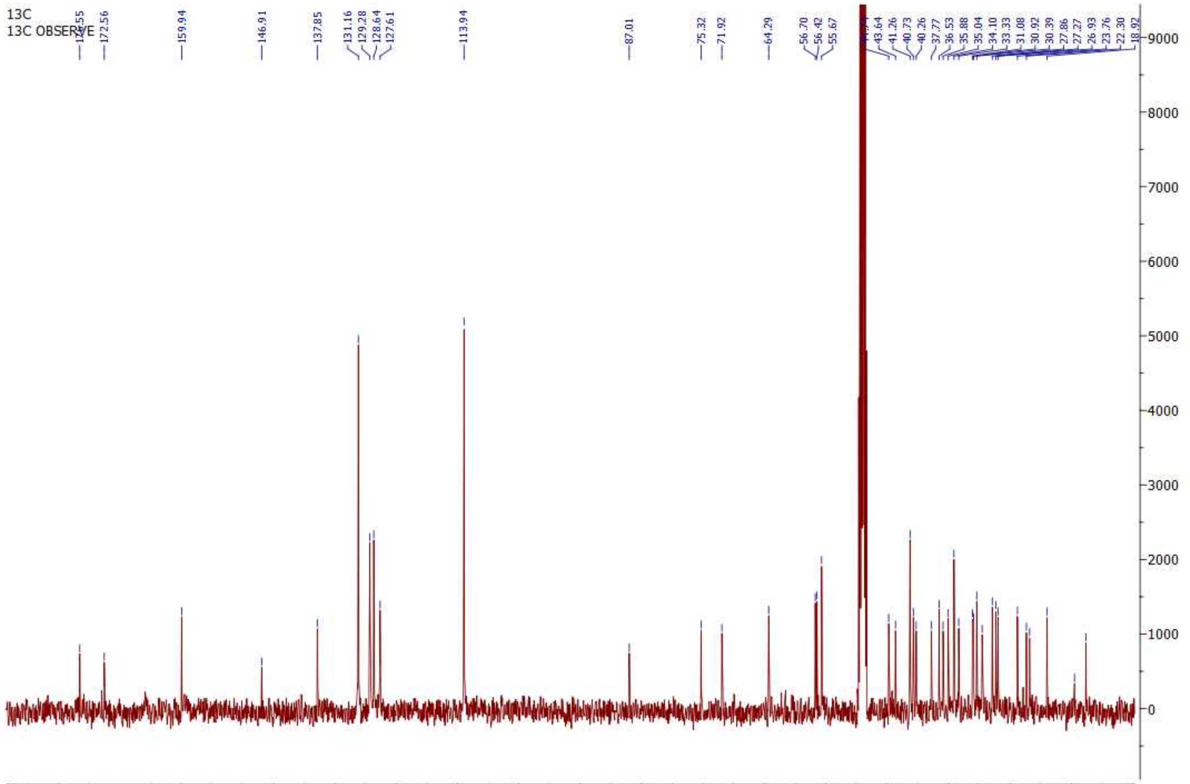

Compound 3: UDC-aminohexyl-alcohol

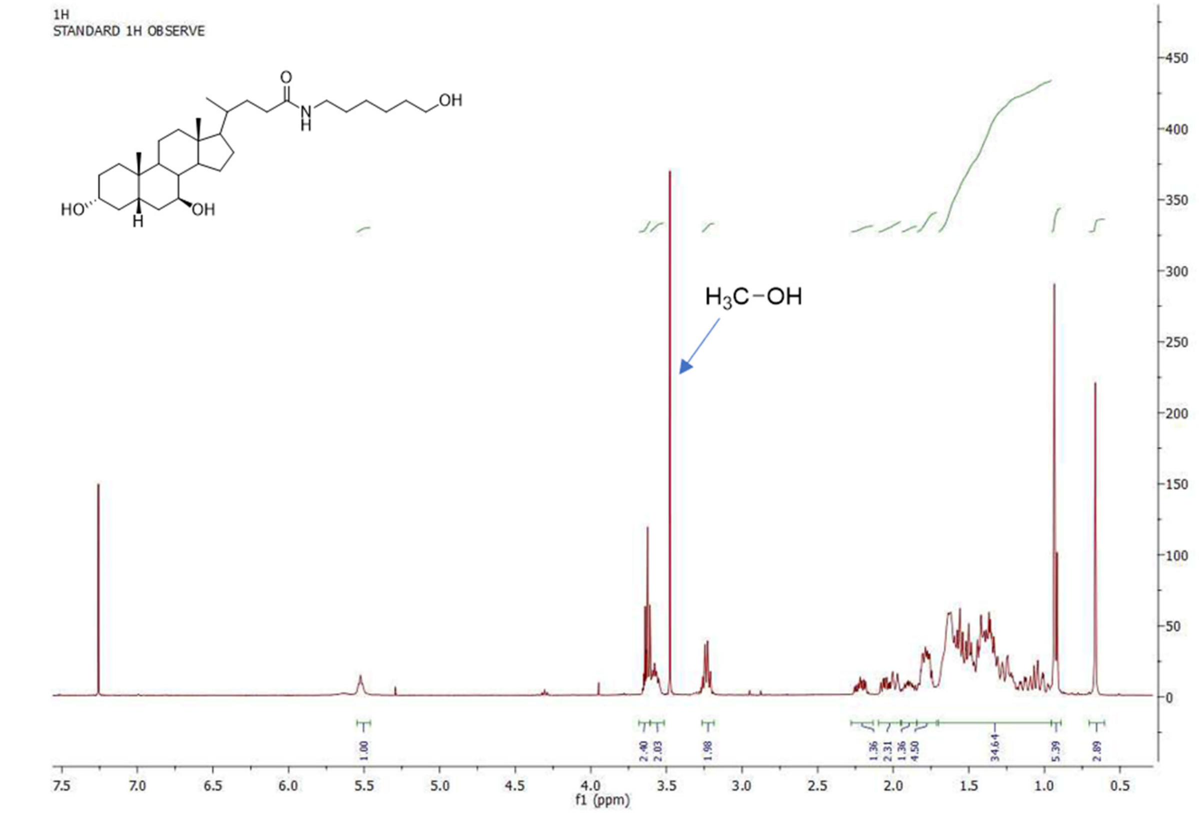
1H
STANDARD 1H OBSERVE
HO
OH
H
O
N
H
OH
H3C−OH
1.00
2.40
2.03
1.98
1.36
2.31
1.36
4.50
34.64
5.39
2.89
f1 (ppm)

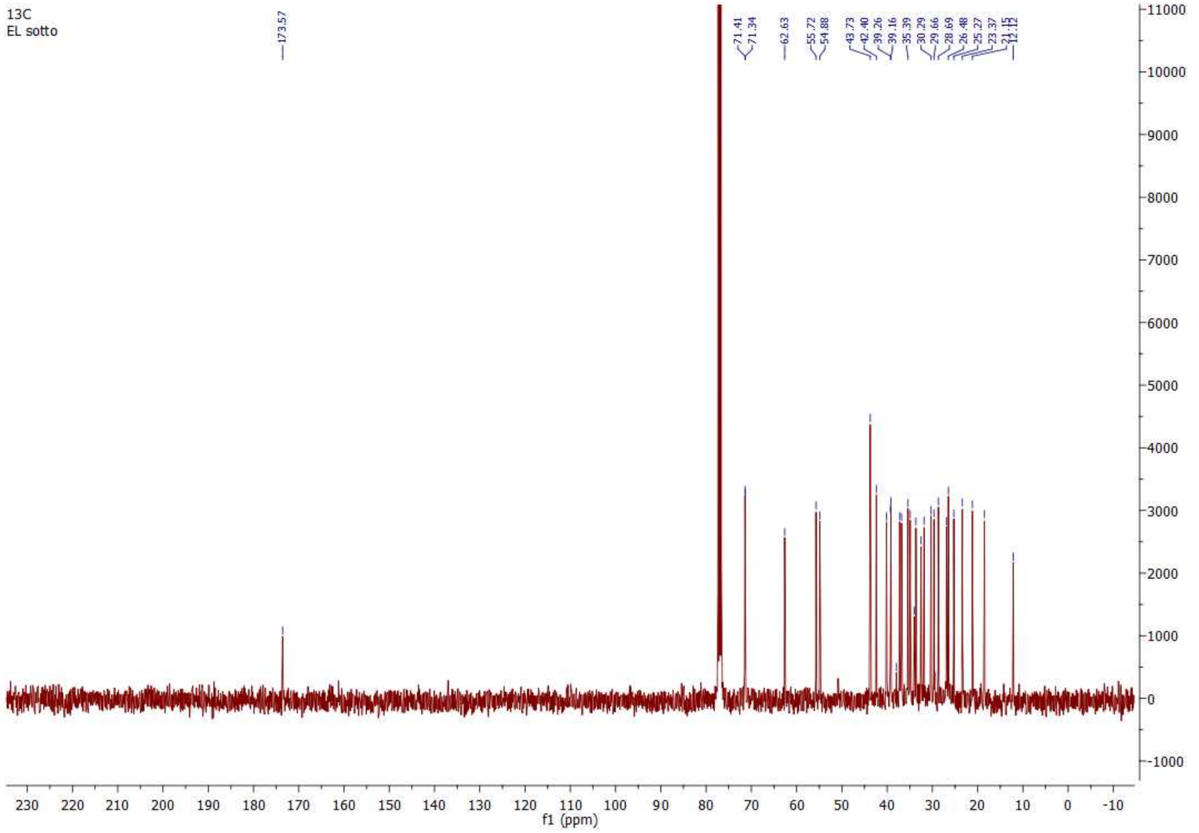
13C
EL sotto
173.57
71.41
71.34
62.63
55.72
54.88
43.73
42.40
39.26
39.16
35.39
30.29
29.66
28.69
26.48
25.27
23.37
21.15
12.12
f1 (ppm)
